# Web-Based Initiatives to Prevent Sexual Offense Perpetration: A Systematic Review

**DOI:** 10.1007/s11920-024-01489-1

**Published:** 2024-03-21

**Authors:** Jana Hillert, Lina Sophie Haubrock, Arne Dekker, Peer Briken

**Affiliations:** Institute for Sex Research, Sexual Medicine & Forensic Psychiatry, Center for Psychosocial Medicine, University Medical Center Hamburg-Eppendorf, Martinistrasse 52, D-20251 Hamburg, Germany

**Keywords:** Web-based, Prevention, Treatment, Sexual violence, Pedophilia, Paraphilia

## Abstract

**Purpose of Review:**

Web-based programs to prevent sexual offense perpetration could provide an opportunity that avoids many of the barriers associated with in-person treatment. The aim of this systematic review is to give an overview of the literature on web-based initiatives aimed at sexual offense perpetration prevention published during the last 10 years (2013–2023) and to report data on the efficacy as well as issues of the discussed initiatives.

**Recent Findings:**

We included 18 empirical studies discussing web-based perpetration prevention initiatives, of which six are randomized controlled trials. Among the articles, four cover programs focusing on prevention of sexual reoffending and 14 discuss programs aimed at preventing initial sexual offenses.

**Summary:**

Evaluations and observations of web-based initiatives aimed at preventing sexual offense perpetration show overall promising results and are well-appreciated. However, evaluation studies are scarce and more randomized controlled trials replicating this effect are warranted.

## Introduction

Sexual offense perpetration is a global public health problem. It is well-established that sexual violence (SV) victimization, of both children and adults, is associated with a multitude of negative psychosocial consequences for the victim as well as for society [1, 2]. According to a meta-analysis of studies from 24 countries, it is estimated that around 8 to 31% of girls and 3 to 17% of boys experience child sexual abuse (CSA) [3]. In recent years, there has been a dramatic increase in the availability of child sexual abuse material (CSAM) [4]. Moreover, approximately one in three women experiences some form of physical or SV over their life course [5]. SV seems to occur especially frequently during college enrollment. Around 25% of female students at US colleges report being sexually assaulted during college [6, 7] and about 9% of female college students report forcible attempted or completed rape [8]. Estimates for prevalence rates of sexual offense perpetration, however, have several limitations. It can be assumed that official reporting data underestimates the actual number of incidents, as it merely considers individuals who are investigated or prosecuted for a criminal offense. This problem is probably particularly pronounced for highly sensitive or stigmatized offenses such as sexual violence and abuse where only a small number of victims report the incidences to the authorities [9, 10]. Given the prevalence and negative impact of sexual violence and abuse, effective prevention is crucial.

Even though there have been calls for a more perpetration prevention centered approach [11], most efforts to prevent sexual offenses either aim to educate potential victims how to protect themselves [12–14] or are embedded in the criminal justice system [11]. Offers for individuals at risk of offending are both scarce and associated with multiple access barriers, including limited offers in rural areas [15]. Men convicted for sexual offenses further list shame, stigmatization, and fear of being reported to the authorities as reasons why they did not seek help prior to their conviction [16]. Besides that, studies show that those convicted for a sexual offense struggle with their sexually deviant thoughts or fantasies several years before their offense [17]. A particular challenge arises for individuals with a pedohebephilic sexual interest, as research indicates biases among health professionals regarding treatment of this group [18]. Taken together, these challenges highlight a gap in the prevention landscape and a need to examine the current status of easily accessible low-threshold prevention offers.

Web-based prevention programs could provide a solution that would avoid many of the barriers associated with in-person treatment offers. They can be accessed from anywhere and at any time, allowing for a high degree of flexibility. An online format can enable anonymity which might lead to more trust and honesty and in turn encourage participation. At the same time, online interventions are not readily comparable to face-to-face programs and need to be assessed for effectiveness. Currently, a wide range of web-based treatment programs are available, and the effectiveness of their application has been supported for several psychiatric disorders (i.e., depression, eating disorders) [19–24]. Results from a meta-analysis of 92 studies examining the efficacy of web-based psychological treatment found effects comparable to in-person treatment [25]. Likewise, a systematic review on 50 studies applying eHealth technologies to the field of forensic psychiatry reports overall promising results. In the majority of the reviewed studies, the web-based programs were at least as effective as in-person interventions targeting the same outcomes [26].

The aim of this systematic review is to provide an overview of the literature on web-based sexual offense perpetration prevention initiatives published during the last 10 years (2013–2023) and to report data on the efficacy as well as issues of the discussed programs. For the purpose of the current review, when discussing prevention offers for sexual offense perpetration, we refer to perpetration of both CSA and SV towards adults. To provide an all-encompassing overview, we also include programs aimed at dynamic perpetration risk factors, such as hypersexuality disorder (HD) [27]. We further include peer-to-peer approaches, as previous research indicates a positive impact on behavioral change and important support in situations of crisis [28]. The aim is to paint a comprehensive picture of the web-based efforts taken in the last 10 years to prevent not only initial sexual offense perpetration but also recidivism, thereby providing a broad overview of the current status of web-based perpetration prevention.

## Method

This systematic review was conducted according to the Preferred Reporting Items for Systematic Reviews and Meta-Analyses (PRISMA) guidelines [29].

### Search Strategy

Studies were searched via the electronic databases PubMed and PsycNet. The searches were concluded in December 2023. We combined search terms relating to web-based prevention programs with search terms regarding sexual offense—CSA and SV—or sexual offense risk factors. The following search terms were used, searching by title and abstract only: (web-based OR internet-based OR online OR computer-based) AND (treatment OR intervention OR therapy OR program* OR psychotherapy OR intervention) AND (sexual abuse OR sexual offence OR sexual offense OR sexual assault OR rape OR sex offend* OR child sexual abuse* OR child pornograph* OR “child sexual abuse material” OR child molest* OR pedophil* OR paedophil* OR “internet sex* offend*” OR minor attracted person* OR paraphilia). We also consulted the publication list of several reviews on the subject as well as relevant program websites. Only studies published 2013 or later were included to focus on most recent developments. In addition, web-based perpetration prevention initiatives without published data were considered.

### Eligibility Criteria

Inclusion criteria were empirical studies (quantitative, qualitative and mixed-method) that discussed web-based programs aimed at preventing sexual offense perpetration, including CSA. Studies in which perpetration prevention was only one of multiple aims were also included. In these cases, we only considered the results regarding sexual offense perpetration, excluding the remaining data. Studies discussing face-to-face, phone-based, or drug-based programs and those published in a language other than English were excluded.

### Selection Process and Data Extraction

Identified records were added to a computerized log in Rayyan.ai [30]. After removing duplicates, screening of title and abstract was conducted independently by the two first authors (JH, LH), making a first selection of relevant articles according to topic relevance and the inclusion and exclusion criteria. At this point, the authors consulted each other to resolve conflicting decisions about first selection before full-text screening those articles. When conflicts arose, the senior author (PB) was consulted until a consensus was reached on inclusion and exclusion.

Data was extracted covering the following topics (if applicable): (a) first author and year of publication, (b) study design, (c) sample characteristics, (d) program name and format, (e) theoretical basis, (h) main outcomes, and (i) main findings.

## Results

### Study Selection

**Fig. 1:**
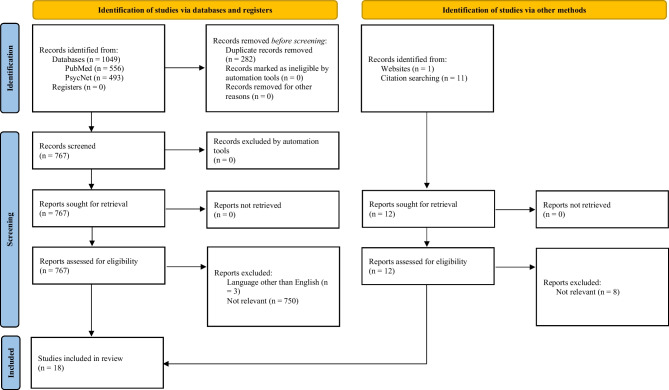
PRISMA 2020 flow chart of included articles

The aforementioned databases were screened with the use of the search terms leading to an identification of 1049 records. After removing 282 duplicates and identifying 12 articles through further methods, 779 articles were screened for abstract and title. This led to an inclusion of 18 articles into the database of this systematic review. An overview of the article selection process is provided in Fig. 1. Moreover, seven further web-based perpetration prevention initiatives without peer-reviewed published data were identified, which will be mentioned cursorily.

### Study Characteristics

Included studies are six quantitative randomized control trials (RCT; 33.33%) [31••, 32•, 33•, 34, 35••, 36•], three qualitative studies (16.66%) [37–39], two experimental-longitudinal studies (11.11%) [40, 41], two single-group intervention studies (11.11%) [27, 42], one between-participant experimental study (5.55%) [43], one quantitative non-randomized study (5.55%) [44], one mixed method study (5.55%) [45], one intervention development study (5.55%) [46], and one RCT study protocol (5.55%) [47]. Attrition rates varied from low [36•] to high [31••, 35••] across studies. Most studies used a mainly Caucasian sample; only one study was conducted in Vietnam [36•], one in Brazil [46], and one with marginalized youths [45]. Moreover, in 14 of the articles included, the authors reported demographics on gender. In 10 of the 14 studies, over 90% of the sample was male [27, 31••, 32•, 33•, 34, 35••, 36•, 39, 43, 44]. The sample characteristics and the study design as well as the main findings can be found in Table 1. As this systematic review aims for a comprehensive picture of all web-based prevention efforts taken, including both the prevention of initial sexual offense perpetration and also of recidivism, it discusses initiatives with somewhat different target groups. Among those articles, four (22.22%) cover web-based perpetration prevention initiatives for individuals who have sexually offended [31••, 32•, 44, 47]. These programs vary from online interventions based on cognitive behavioral therapy (CBT) to emotion regulation interventions. Three of those interventions target individuals who have sexually molested a child [31••, 44, 47], whereas one targets men with a sexual offense perpetration history, not necessarily with regard to children [32•]. The 14 remaining articles (77.77%) discuss perpetration prevention programs for non-offending individuals who are sexually attracted to children [37, 38], for college students [33•, 34, 35••, 36•, 40–43, 45, 46], for youths [43, 45], or for individuals fulfilling the criteria for hypersexuality disorder [27]. It is worth noting that two perpetration prevention programs for college students (*RealConsent* and *KisS*) are respectively discussed in two articles [33•, 35••, 40, 41].

**Table 1:** Design, sample characteristics, program information, and main findings of included studies

First author	Study design	Sample characteristics	Program name and format	Theoretical basis	Main outcomes	Main findings
Fromberger et al. [47]	Study protocol (RCT)	*N* = 428 subjects expected to complete the intervention; male and female individuals under community supervision for CSA or CSAM use	*@myTabu*; six modules targeting different risk factors (self-guided and coach-guided); monetary compensation	RNR approach with CBT techniques	Officially recorded re-offenses and dynamic risk factors	Ongoing evaluation
Lätth et al. [31••]	RCT	*N* = 160; adult CSAM users, 99% male	*Prevent It*; eight modules targeting themes related to CSAM viewing; psychotherapist feedback	CBT	Self-reported time spent using CSAM during the past week	Significantly larger decrease in weekly CSAM viewing time in the *Prevent It* group compared to Placebo participants
Schuler et al. [44]	Cross-sectional study	*N* = 4161; individuals with sexual interest in prepubescent and early pubescent children; 90.9% male	*Troubled Desire* App; self-assessment and self-management training modules; available in seven different languages	Berlin Dissexuality Therapy Program	Sexual interest and offense characteristics	78.9% reported a sexual interest in children; these participants were more likely to experience distress and trouble as well as commit CSA and use CSAM; the majority of offenses was not reported to legal authorities
Rask Pedersen [37]	Ethnographic observations and sampling	Anonymous non-offending pedophiles	*Virtuous Pedophiles*; peer support forum	n/a	n/a	Non-offending pedophiles should not identify as monsters; individuals are not responsible for their thoughts but can control their actions
Jones et al. [38]	Qualitative	Anonymous non-offending pedophiles; 196 unique extracts	*Virtuous Pedophiles*; peer support forum	n/a	n/a	Three overarching themes were identified: accepting pedophilic interests, coping strategies in risk situations, substitutions to satisfy their sexual needs
Bekkers et al. [39]	Qualitative	*N* = 15; moderators, members, mental health professionals involved in a Dutch community for individuals with a sexual interest in children; mostly male	Dutch peer support forum for individuals with a sexual interest in children including three chat rooms	n/a	n/a	Improved self-acceptance, decreased sense of loneliness, better coping with stigma and less psychological distress, possibility for moderators to set moral boundaries regarding respect for children
Davis et al. [32•]	RCT	*N* = 209; men with sexual assault perpetration history; mean age = 24.76	Brief ER-focused interventions (five-to-10-min)	CR and mindfulness	Sexual aggression, emotion regulation skills, anger, sexual arousal, coercive tactic intentions, non-consensual sex intentions	Both CR and mindfulness intervention resulted in less non-consensual sex intentions compared to control; the CR intervention compared to the mindfulness intervention resulted in less coercive tactic intentions and less non-consensual sex intentions in intoxicated men with a severe perpetration history
Gilmore et al. [42]	Pilot study	*N* = 24; undergraduate students who engaged in heavy episodic drinking last month; 33% cisgender heterosexual men, 38% cisgender heterosexual women, 29% LGBTQ; mean age = 19.63	+ *Change*; brief intervention that targets alcohol misuse, sexual assault perpetration, bystander behaviour, and sexual assault risk reduction; mean completion time: 17.52 min	Motivational interviewing	Alcohol use, sexual assault victimization risk, sexual assault perpetration risk, bystander behavior	After + *Change* participants were significantly more conscious of the problem of sexual perpetration and showed greater willingness to make changes to prevent sexual offense perpetration
Salazar et al. [35••]	RCT	*N* = 743; male college students; mean age = 20.38	*RealConsent*: six 30-min modules, self-paced, to be completed within three weeks, monetary compensation	Social cognitive theory, social norms theory and bystander educational model	Perpetration and bystander behavior	*RealConsent* intervention significantly reduced SV perpetration; this change was sustained over six-month follow-up; the odds for perpetrating among *RealConsent* participants were 73% lower than of participants in the comparison condition
Salazar et al. [33•]	RCT	*N* = 743; male college students; mean age = 20.38	*RealConsent*: six 30-min modules, self-paced, to be completed within three weeks, monetary compensation	Social cognitive theory, social norms theory, and bystander educational model	Perpetration and bystander behavior	*RealConsent* intervention significantly reduced SV perpetration; effect of *RealConsent* on SV perpetration was mediated by legal knowledge of assault/rape, knowledge of effective consent for sex, rape myth acceptance, and date rape attitudes
Yount et al. [36•]	RCT	*N* = 793; heterosexual and bisexual male college students in Vietnam; mean age = 18	*GlobalConsent*: six 30-min modules, self-paced, to be completed within 12 weeks, monetary compensation	Social cognitive theory, social norms theory and bystander educational model; based on *RealConsent*	Perpetration and bystander behavior	Men in the *GlobalConsent* group had lower odds of engaging in sexually violent behavior compared to men in the AHEAD control group
Schuster et al. [40]	Experimental four-wave longitudinal study	*N* = 1181; German university students; 762 female, 419 male; mean age = 22.6	*Competence in sexual situations* (*KisS*): program to reduce risk and vulnerability factors of sexual aggression perpetration and victimization; six modules in weekly intervals	Theory of sexual scripts and social learning theory	Behavioral risk and vulnerability factors of sexual aggression	Risky sexual behavior at both follow-up points was lower in the intervention than in the control group, via less risky sexual scripts one week after the program; participants in the intervention group showed less risky sexual scripts compared to the control group
Tomaszewska et al. [41]	Experimental four-wave longitudinal study	*N* = 1181; German university students; 762 female, 419 male; mean age = 22.6	*Competence in sexual situations* (*KisS*): program to reduce risk and vulnerability factors of sexual aggression perpetration and victimization; six modules in weekly intervals	Theory of sexual scripts and social learning theory	Sexual aggression victimization and perpetration	Indirect effects of the intervention on sexual aggression perpetration were reported (mediated by risky sexual scripts and risky sexual behavior)
Wong et al. [34]	RCT	*N* = 241; heterosexual male college students; mean age = 20.26	Brief intervention (45 min) to reduce sexual aggression perpetration	Self-persuasion	Self-perceived likelihood of sexual aggression, sexual aggression perpetration, bystander behavior, in-group solidarity	Participants in the self-persuasion condition reported lower levels of self-perceived likelihood of committing sexual aggression after the intervention; however, at six months follow-up, there was no difference between intervention and control groups
Murta et al. [46]	Intervention development study	*N* = 1220 Brazilian youths expected to participate	*Dating SOS*: guided online intervention to prevent dating violence and improve romantic relationship quality; four sessions (15 min each)	I-Change Model; attachment theory	Attachment style, conflict resolution skills, victimization and perpetration of psychological, physical, and/or sexual violence in relationships, relationship quality	Ongoing evaluation
Segura et al. [45]	Mixed-method triangulation design: EMA, post survey, semi-structured interviews	*N* = 39 Latinx/Hispanic, Asian American, Arab American, and African American youths; mean age = 16.7	*DOT* (*Dream, Own & Tell*): 13-to-18-week SV prevention program; eight-to-10-session educational and leadership course in weekly intervals and social media campaign; five-to-eight-week community mobilization practicum; monetary compensation	Situational cognitive model of SV and DV prevention and diffusion of innovation theory	Acceptability and experience of online program	94.4% were satisfied with participation; 83.3% agreed that online format gave them the same quality of knowledge and skills as face-to-face learning; 50% of participants found the accessibility and convenience convincing, overall positive feedback in interviews
Nicolla et al. [43]	Between-participant experimental design	*N* = 580 adolescents; 97% male; mean age = 17.3	Personal narrative videos discussing SV on TikTok	Social norms theory	Objective knowledge of consequences, perceived severity, perceived knowledge of consequences, willingness to talk with peers, attention, empathy, relevance, reactance	Personal narrative TikToks about SV led to higher objective knowledge of the consequences of SV and higher perceived severity of SV compared to control TikToks
Hallberg et al. [27]	Pilot case report	*N* = 36; male adults who fulfil criteria for HD; mean age = 39	10 modules over 12-week period; each participant had a personal therapist	CBT	Hypersexuality, severity of HD symptoms, sexual compulsivity, self-rated severity of paraphilic disorder, psychological distress	Therapist-assisted iCBT led to significant decrease in hypersexual symptoms which was sustained three months after completion

*RCT* randomized controlled trial, *CSA* child sexual abuse, *CSAM* child sexual abuse material, *RNR* risk-need-responsivity, *CBT* cognitive behavioral therapy, *iCBT* internet-delivered cognitive behavioral therapy, *ER* emotion regulation, *CR* cognitive restructuring, *SV* sexual violence, *HD* hypersexuality disorder, *AHEAD* attention-control adolescent health education, *EMA* ecological momentary assessment, *DV* dating violence

### Web-Based Initiatives for Individuals Who Have Sexually Offended—Target Group, Content, and Efficacy

As mentioned above, four studies discuss web-based perpetration prevention initiatives for individuals who have sexually offended. For only two of the four initiatives, an effectiveness evaluation is published. Lätth et al. [31••] conducted an RCT (single-blinded participants, parallel-group, superiority, psychological placebo-controlled) with active CSAM users (18 +) who took part in an anonymous online intervention called *Prevent It*, developed by the Karolinska Institutet in Stockholm. Notably, this RCT (pilot) was the first of its kind in this field. *Prevent It* is a CBT-based program in English consisting of eight modules related to the use of CSAM. The primary outcome was the self-reported time spent watching CSAM within the last week, assessed by the SChiMRA + measure [48]. Compared to the placebo control group, participants in the *Prevent It* group showed a significant decrease in CSAM viewing time. The program was well-appreciated by participants who reported hope and a heightened awareness of their thoughts and behaviors.

*Troubled Desire* is an anonymous app-based perpetration prevention program for individuals who are sexually attracted to children [44]. The app consists of self-assessment and self-management training modules and is available in German, English, Spanish, Portuguese, French, Hindi, and Marathi. There is no published data on its effectiveness. Descriptive analysis of the self-assessment data revealed that 78.9% of the sample had a sexual interest in children. It is however unclear how this percentage came about, as the target groups of *Troubled Desire* are individuals with a sexual interest in children. Individuals who offended and who reported sexual interest in children were more likely to experience distress as well as report CSAM use vs. individuals who sexually offended with no interest in children.

The target group of a third web-based intervention, called *@myTabu*, are individuals (18 + ; males and females) under community supervision for CSA or CSAM use. The program is currently being evaluated, and so far, only the study protocol for the RCT is available, while the results are still pending [47]. The program consists of six predefined modules that target diverse dynamic risk factors with coach-guided feedback. Both conditions (intervention vs. placebo) include 24 sessions to be completed in 24 weeks. The program is based on the risk-need-responsivity (RNR) approach [49] and CBT techniques. A monetary reward system is included to motivate participants. To assess efficacy of this intervention, recidivism (recorded re-offenses) and self-report questionnaires on empirically supported dynamic risk factors will be assessed for both groups.

While the previously described initiatives (*Prevent It*, *Troubled Desire*, and *@myTabu*) target individuals who are sexually attracted to children, Davis et al. [32•] tested a program addressing men with a history of sexual perpetration against adults who reported at least one incident of sexual coercion in their lifetime. The study used an RCT design to test how alcohol intoxication influences two different five-to-10-min emotion regulation interventions (cognitive restructuring (CR) and mindfulness) that aim to reduce sexual aggression compared to an attentional control condition. Participants were blinded to all conditions and were told that they would practice coping strategies for negative emotions. Results showed that the CR intervention as well as the mindfulness intervention resulted in less non-consensual sex intentions compared to control. This effect was particularly pronounced for the CR group. Notably, for intoxicated men with a more severe perpetration history, the mindfulness condition led to higher coercive tactic intentions compared to individuals in the control and CR condition.

### Web-Based Initiatives for Non-offending Individuals—Target Group, Content, and Efficacy

These next studies included in this review target primary perpetration prevention. As previously mentioned, two prevention programs—*RealConsent* and *KisS* (*competence in sexual situations*)—are respectively discussed by two articles [33•, 35••, 40, 41]. *RealConsent* targets perpetration prevention and is an intervention based on social cognitive theory, social norms theory, and the bystander educational model. The intervention focuses on contents such as sexual consent, rape myth, norms regarding gender roles, effective communication, alcohol and rape, victim empathy, and bystander behavior. It comprises six modules which last 30 min each and can be completed within three weeks. A third article discusses a further expansion (from three to 12 weeks) and cultural adaptation of *RealConsent*, called *GlobalConsent* [36•]. Both programs helped to reduce SV perpetration [33•, 35••, 36•]. The effect of *RealConsent* seems to be mediated by four factors [33•]: knowledge of effective consent for sex, hostility towards women, date rape attitudes, and hypergender male ideology. Even though it was reported in both studies that the effect sustained over a follow-up period, Yount et al. [36•] summed the results of posttests 1 (after six months) and 2 (after 12 months), making it impossible to determine the actual effect at the respective time points. Besides this, there was no posttest immediately after the intervention. *KisS* aims to reduce risk and vulnerability factors of sexual aggression perpetration and victimization. Based on sexual scripts and social learning theory, it is designed as a six-week prevention program with weekly modules. Compared to the control group, risky sexual behavior was lower in the intervention group nine and 12 months after the intervention, indicating a long-lasting impact. The impact on risky sexual behavior at nine and 12 months was mediated via less risky sexual scripts one week after the end of the program [40]. Tomaszewska et al. [41] additionally reported indirect effects of the intervention on sexual aggression perpetration: the impact at nine and 12 months was mediated via less risky sexual scripts one week after the program and risky sexual behavior nine months after the intervention [41].

Further web-based initiatives, *Dating SOS*, introduced by Murta et al. [46], and *DOT* (*Dream, Own & Tell*), discussed by Segura et al. [45], aim at preventing dating violence (DV) in youths. *Dating SOS* is a guided web-based program for Brazilian youths consisting of individual sessions where participants learn about different aspects linked to romantic relationships, such as victimization and perpetration of DV. Subsequently, they receive personalized feedback via text message. An effectiveness and usability evaluation (RCT) of the program is ongoing. Project *DOT* consists of three core components, an “Educational and Leadership Curriculum” in group sessions on Zoom, development of a social media campaign, and a community mobilization practicum [45]. Pre-COVID-19, the program was administered in-person but was adapted to an online format due to the pandemic. This online version was found to be valuable and was perceived as a safe space by participants. In a between-subject experimental study with youths, Nicolla et al. [43] used existing personal narrative videos on TikTok (a social media platform for viewing and sharing short videoclips) discussing SV to prevent SV perpetration through conveying personal experience. Viewing led to more information on consequences of SV and higher perceived severity of SV compared to control.

In an RCT, Wong et al. [34] tested a 45-min brief intervention aimed at reducing sexual aggression perpetration of heterosexual male college students. The study revealed that participants in the self-persuasion intervention condition, which addressed sexual aggression, reported decreases in self-perceived likelihood of committing sexual aggression after the intervention. However, this effect was not maintained until the six-month follow-up assessment.

In a pilot study, Gilmore et al. [42] tested the + *Change* initiative, another brief intervention. The program targets alcohol misuse, sexual assault perpetration, bystander behavior, and sexual assault risk reduction. On average, participants took 17.52 min to complete the program. The findings revealed that after the program, participants were significantly more conscious of the problem of sexual perpetration and showed greater willingness to make changes to prevent sexual assault perpetration.

In the last study discussing a primary prevention initiative—a pilot study by Hallberg et al. [27]—participants were not college students or youths, but individuals who fulfill the criteria for hypersexuality disorder (HD). HD is associated with recurrent sexual thoughts and behaviors, which can be a risk factor for recidivism in sexual offenses [39]. A quarter of participants fulfilled at least the criteria for one paraphilic disorder or reported paraphilic thoughts. In this uncontrolled study, participants underwent an intervention to reduce hypersexual symptoms. The intervention consisted of 10 modules distributed over a 12-week period. The intervention led to a significant pre-post decrease in hypersexual symptoms which was sustained three months after completion.

### Peer-to-Peer Support Forums for Non-offending Individuals Who Are Sexually Attracted to Children

Among the 18 included articles, three are qualitative studies that cover topics discussed by non-offending individuals who are sexually attracted to children in the peer support forum *Virtuous Pedophiles* [37, 38] and a Dutch peer support forum [39]. *Virtuous Pedophiles* has more than 4700 members and offers help for those with a sexual interest in children who report that they are living offense-free. The forum has strict rules about topics of discussion and can be monitored by the Police. Jones et al. [38] identified three predominant themes of discussion by applying thematic analysis to 326 forum posts: accepting pedohebephilic interest, coping strategies in risk situations, and substitutions to satisfy sexual needs. In an ethnographic study about the same online forum, Pedersen [37] concludes that peer-to-peer support seems to be a relevant resource for a fulfilling life by reducing fear to seek help. He states that non-offending pedophiles should not define themselves as monsters and individuals are not responsible for their thoughts and are able to control their actions. Thus, one might assume an increased willingness to seek help from professionals and thereby prevent sexual offenses. The Dutch forum is an online community of individuals with a sexual interest in children. It consists of three chat rooms and had over 600 members in the last five years. Bekkers et al. [39] analyzed 15 interviews with members, moderators, and mental health professionals involved in the forum and identified that peer support can lead to increased self-acceptance and coping with stigma as well as less loneliness and psychological distress of members. The authors conclude that online communities can have a positive impact on behavioral change and well-being.

### Additional Perpetration Prevention Initiatives

At present, diverse web-based programs are being developed and evaluated for individuals at risk of committing CSA. Among those that are already implemented but still under evaluation are *@myTabu*, *Troubled Desire*, and *Project Bridge* [50], an adapted version of *Prevent It* [51] as well as *HelpWanted* [52]. *Project Bridge* consists of the five-week CBT-based self-help program *ReDirection* as well as motivational interviewing (*Mi Bridge*), which both aim to deliver strategies to stop CSAM use. It is available in English, Finnish, Spanish, Czech, Slovak, and German. *Prevent It* [51] has been revised since the first version and now consists of nine modules and targets individuals with sexual urges towards children, regardless of whether they have offended in the past or not. The program is available in German, Swedish, and Portuguese as well as in English. *HelpWanted* [52] is a five-module online course for adolescents and young adults who are sexually attracted to younger children, aiming to support participants in living a safe and non-offending life. Further available resources comprise the self-help website of *StopItNow! UK & Ireland* [53] (including *Get Support* [54] and *Get Help* [55]) from the Lucy Faithful Foundation, which offers educational material as well as a live chat for individuals worried about their sexual thoughts about children or their online behavior. *StopItNow!* offers another program which specifically targets juveniles, called *What’s OK* [56], which provides information on sexually safe behaviors and a live chat.

## Discussion

The present systematic review gives an overview of recent developments of web-based sexual offense perpetration prevention initiatives. Web-based programs can provide a low-threshold opportunity for individuals who seek help, particularly for those scared of being stigmatized. For instance, studies have shown that men who sexually offended struggle with their thoughts up to 10 years before committing an offense, emphasizing the need for further help services [17]. Taken together, all evaluation studies reported in this review support that web-based initiatives to prevent sexual offense perpetration can be considered a good low-threshold alternative or addition to face-to-face treatments and in-person support groups.

This review included 18 articles which cover web-based perpetration prevention initiatives from the last 10 years. In sum, all interventions aim to prevent SV perpetration, sometimes in a narrower and sometimes in a broader sense. The six included RCTs revealed encouraging results in support of web-based initiatives for prevention of initial sexual offense perpetration as well as recidivism. Three were especially valuable for this review, as they tested module-based programs. They will be discussed using the six quality criteria Schröder et al. [57] developed to evaluate web-based programs for individuals at risk of sexually offending against children: “Evaluation if, why and for whom the program achieves its goal”; “Testing of cost-effectiveness”; “Examination for negative side effects”; “Voluntary participation”; “Selection of an appropriate control group”; and “Conducting the evaluation in a secure environment”. One of the RCTs found that CSAM viewing time decreased after the eight-week therapist-guided CBT-based *Prevent It* intervention [31••]. Even though the effect was small, it was significant. The intervention fulfils five of the six quality criteria, hence indicating a scientifically well-established intervention and further underlining the relevance of this study. According to Schröder et al. [57], the six evaluation criteria could further be applicable to web-based interventions with different target groups. The evaluations of *RealConsent* [33•, 35••] and *GlobalConsent* [36•] adhere to four of the six criteria, providing further support for these programs. The studies yielded comparable results in that the intervention helped college men to reduce sexually violent behavior. Due to the cultural differences between these samples, one could assume that this effect may generalize to other populations as well. It would be interesting to see future studies on sexual offense perpetration prevention, working with the criteria developed by Schröder et al. [57] for the evaluation of their initiatives. Even though Davis et al. [32•] and Wong et al. [34] did not evaluate module-based programs and hence cannot be fully compared to the three RCTs mentioned above in terms of content and scope, the studies provide support that brief web-based perpetration prevention initiatives may be effective in reducing sexual offense recidivism as well as initial offenses. However, in the brief intervention by Wong et al. [34], the effect was not sustained six months after the intervention indicating that brief interventions might be limited to short-term effects. Overall, these findings suggest the usefulness of web-based perpetration prevention, which is in line with prior studies supporting the effect of web-based interventions for different target groups [19–26]. It seems they provide low-threshold opportunities, especially for young adolescents. Ultimately, the informative value of these few evaluations reporting this positive effect is limited and more evaluation studies following this most rigorous design are needed.

Other web-based initiatives aiming at perpetration prevention include peer-to-peer support forums. Even though they cannot be considered an intervention based on therapeutic principles in a narrower sense, they can function as a positive environment and might have a positive impact on behavioral change as well as provide support in situations of crisis [37–39]. Thus, they can function as an alternative assistance for individuals concerned about their risk to sexually offend.

Certain strengths and limitations of the studies included should be mentioned that impact the informative value of this review. Notably, participants in *RealConsent*, *GlobalConsent*, *KisS*, and *Prevent It* were asked about their actual behavior and not only their behavioral intentions, which can be considered a strength of these studies. Nevertheless, the results of this review should be interpreted with caution due to the few evaluations conducted using an RCT, the most rigorous statistical design to test effectiveness. Furthermore, there was a high attrition rate in some of the studies included, as well as some further methodological issues, such as small samples or the lack of a control group. Most studies relied on self-report and are thus prone to socially desirable responses, especially regarding criminal behavior [58], even though guaranteed anonymity could have negated this effect. More research is needed to further test web-based initiatives in an evidence-based manner, as evaluations are still scarce. However, the ongoing trials mentioned in this review could help close this knowledge gap. In the future, it would be interesting to compare the impact of web-based programs to in-person treatment offers for individuals at risk of committing a sex offense. Additionally, therapist-guided web-based programs show more promising results in terms of effectiveness compared to self-guided treatment [22]. Thus, it will be interesting to compare these two formats for web-based perpetration prevention.

## Limitations

The present review has several limitations. Reviewed papers were limited to the last 10 years and thus, we might have excluded articles relevant to this topic. Moreover, only few studies were available due to the relative novelty of the subject discussed. Therefore, there was not enough quantitative data to enable a meta-analysis. Additionally, most studies used Caucasian samples and only few programs were tested in non-Western populations; hence, it is unclear whether the results generalize to other cultures as well. Lastly, it is noteworthy that most studies included used samples in which over 90% of participants were male.

## Conclusion

The presented evaluations of web-based initiatives revealed promising results in targeting perpetration prevention and additionally received positive feedback from participants. Nonetheless, high victimization prevalence rates remain a problem and this review further underlines the need for more development and rigorous testing of new initiatives in the field. We are confident that the current ongoing evaluations will be able to further clarify the potential of web-based programs to prevent sexual offense perpetration.

## Declarations

### Disclaimer

The content of this publication represents the views of the authors only and is their sole responsibility. The European Commission does not accept any responsibility for use that may be made of the information it contains.
